# A phase Ib study of everolimus combined with metformin for patients with advanced cancer

**DOI:** 10.1007/s10637-017-0478-4

**Published:** 2017-06-15

**Authors:** Remco J. Molenaar, Tim van de Venne, Mariëtte J. Weterman, Ron A. Mathot, Heinz-Josef Klümpen, Dick J. Richel, Johanna W. Wilmink

**Affiliations:** 1 Department of Medical Oncology, Division of Internal Medicine, Academic Medical Center, University of Amsterdam, Meibergdreef 15, 1105 AZ Amsterdam, The Netherlands; 2 Department of Medical Biology, Academic Medical Center, University of Amsterdam, Meibergdreef 15, 1105 AZ Amsterdam, The Netherlands; 3 Cancer Center Amsterdam, Location Academic Medical Center, University of Amsterdam, Meibergdreef 15, 1105 AZ Amsterdam, The Netherlands; 4 Department of Clinical Pharmacology, Academic Medical Center, University of Amsterdam, Meibergdreef 15, 1105 AZ Amsterdam, The Netherlands

**Keywords:** Everolimus, Metformin, Cancer, Pharmacokinetics, Safety, Toxicity

## Abstract

*Background* The efficacy to monotherapy with the mTOR inhibitor everolimus in advanced cancer is often limited due to therapy resistance. Combining everolimus with metformin may decrease the chance of therapy resistance. *Methods* Patients received everolimus and metformin in a 3 + 3 dose-escalation scheme. Objectives were to determine the dose-limiting toxicities (DLTs), maximum tolerated dose, toxic effects, pharmacokinetics and anti-tumour efficacy. *Results* 9 patients received study treatment for a median duration of 48 days (range: 4–78). 6 patients discontinued due to toxicity and 3 patients because of progressive disease. At the starting dose level of 10 mg everolimus qd and 500 mg metformin bid, 3 out of 5 patients experienced a DLT. After de-escalation to 5 mg everolimus qd and 500 mg metformin bid, considerable toxicity was still observed and patient enrollment was terminated. In pharmacokinetic analyses, metformin was eliminated slower when co-administered with everolimus than as single-agent. After 9 weeks of treatment, 3 patients were still on study and all had stable disease. *Conclusion* The combination of everolimus and metformin is poorly tolerated in patients with advanced cancer. The pharmacokinetic interaction between everolimus and metformin may have implications for diabetic cancer patients that are treated with these drugs. Our results advocate for future clinical trials with combinations of other mTOR inhibitors and biguanides.

**Electronic supplementary material:**

The online version of this article (doi:10.1007/s10637-017-0478-4) contains supplementary material, which is available to authorized users.

## Introduction

Many cancers display activation of the phosphatidylinositol 3-kinase (PI3K)/protein kinase B (AKT)/mammalian target of rapamycin (mTOR) pathway, which plays an important role in controlling the proliferation and metabolism of cancer cells [1]. However, only few malignancies demonstrate clinical response to mTOR inhibitors, such as everolimus. Accordingly, mTOR inhibitors are only FDA- and EMA-approved for use in patients with a few types of cancer, such as mantle cell lymphoma, pancreatic neuroendocrine tumors, advanced renal cell carcinoma, and giant cell astrocytoma [2–6].

In other solid organ malignancies, mTOR inhibitors show clinical responses, though in most cases not enough to warrant monotherapy and with considerable toxicity. A major issue is that most cancers develop resistance against monotherapy with mTOR inhibitors. One of the proposed mechanisms how tumor cells develop resistance to mTOR inhibition is through the mTOR-dependent negative feedback loop that acts to inhibit PI3K/AKT activity. Via this mechanism, drugs that target mTOR inhibit neoplastic processes downstream of mTOR but also the feedback loop. Subsequently, this may activate AKT [7] and its downstream oncogenic effects [8], thereby limiting the efficacy of monotherapy with mTOR inhibitors [9, 10].

A possible way to prevent the development of therapy resistance against mTOR inhibitors is by combining these drugs with the mitochondrial respiration inhibitor metformin, a drug commonly prescribed for the treatment of type 2 diabetes mellitus (T2DM). Several in vitro studies that combined metformin with cytotoxic agents or with everolimus have shown a synergistic inhibition of breast cancer cell growth, compared to single-agent therapy [11, 12]. Metformin’s synergistic antiproliferative effect in combination with everolimus is a result of activation of 5′ adenosine monophosphate-activated protein kinase (AMPK), which inhibits AKT. Via this mechanism, metformin counteracts the oncogenic AKT activation that is induced by mTOR inhibition [13, 14]. In short, because metformin may delay or overcome the mechanism of treatment resistance against monotherapy with an mTOR inhibitor, there is a rationale for combining these two drugs [7]. In a retrospective combined analysis of 94 patients from three clinical trials with various mTOR inhibitors for the treatment of endometrial cancer, self-reported metformin use was associated with a higher response rate to an mTOR inhibitor (18 vs. 7%) and a lower rate of disease progression (12 vs. 33%) [15]. Also, a clinical trial combining everolimus and letrozole for the treatment of endometrial cancer found that patients that received metformin to treat hyperglycemia (either in the context of pre-existing diabetes or study treatment-related) had a significantly higher response rate (56 vs. 23%) [16]. Moreover, metformin has attracted interest as an anti-cancer drug [17] since an association between metformin use in T2DM patients and a reduced risk of breast, colon, pancreas and prostate cancer was acknowledged [18–23], as well as a reduced risk of mortality, as compared with patients treated with insulin or sulfonylureas [24].

The present clinical trial investigates the safety and maximum tolerated dose (MTD) of a combination therapy with everolimus and metformin in patients with advanced cancer. As secondary objectives, the pharmacokinetics of everolimus and metformin combination treatment and tumor responses to study treatment were assessed.

## Materials and methods

### Patient population

Eligible patients were aged ≥18 years with histological or cytological confirmed solid malignancies that were refractory to standard therapies, or for which no standard treatment option was available. Other eligibility criteria included WHO performance status ≤2, estimated life expectancy of ≥3 months, adequate bone marrow (white blood cell count ≥3.0 × 10^9^/l, platelets ≥100 × 10^9^/l), hepatic (serum bilirubin ≤1.5 × upper limit of normal (ULN), ALAT/ASAT ≤2.5 × ULN or in case of liver metastases ≤5 × ULN) and renal function (serum creatinine ≤150 μmol/l). Patients also had to be mentally, physically and geographically able to undergo the study treatment and follow-up. Patients were ineligible if they were known with a serious concomitant medical condition, such as unstable angina pectoris, symptomatic congestive heart failure, myocardial infarction within the last 6 months before screening, serious uncontrolled cardiac arrhythmia, uncontrolled diabetes (fasting serum glucose >2 × ULN), severe infection, cirrhosis, chronic active/persistent hepatitis or severly impaired lung function. Furthermore, patients were ineligible when they had a known hypersensitivity to either study drug, if they had used either study drug in the previous 6 months or when they were concomitantly treated with strong CYP3A4/3A5/2C8 inhibitors/inducers. Women were ineligible when they were pregnant or lactating. All patients gave written informed consent.

### Medical ethics

The study protocol was approved by the Medical Ethics Committee of the Academic Medical Center (reference number NL_37906.018.12) and was conducted in accordance with the Declaration of Helsinki and Good Clinical Practice guidelines.

### Study design

This was a phase I, open-label, single-center, dose escalation study to assess the safety, DLTs, maximum tolerated dose (MTD) and the pharmacokinetic interaction of the combination of everolimus and metformin. The study was conducted at the Academic Medical Center (The Netherlands). At least three patients per dose level were recruited and the cohort at this dose level was to be expanded to six patients if 1/3 patients experienced a DLT. Dose escalation to the next dose level was to be permitted if no DLT occurred in 0/3 or in ≤1/6 patients. In case of DLT(s) in ≥1/3 or in ≥2/6 patients, that dose level was to be declared intolerable and dose-escalation occurred. If dose level −1 was also intolerable, the study was to be terminated without establishment of an MTD. A DLT was defined as any of the following events related to study treatment and occurring during the first treatment cycle, defined by National Cancer Institute Common Terminology Criteria for Adverse Events version 3.0 (CTCAE): neutropenia grade 4 lasting >5 days or febrile neutropenia grade 3 (fever ≥38.5 °C), grade ≥3 anemia, grade ≥4 trombocytopenia or grade 3 trombocytopenia with bleeding, vomiting grade ≥2, diarrhoea grade ≥2 or any other toxicity grade ≥3 (excluding alopecia), despite optimal supportive care. In case of measurable disease, tumor measurements were performed using a CT scan at baseline and every nine weeks and were evaluated in accordance with the Response Evaluation Criteria in Solid Tumors (RECIST 1.1) [25].

### Study treatment

During the first seven days of treatment, patients were treated with single-agent everolimus to reach steady-state concentrations. Treatment with metformin bid started on day 8. The everolimus and metformin doses were to be de-escalated or escalated as shown in Table 1. Metformin was administered bid instead of qd at dose level 1 to mimic conventional metformin dosing schedules in T2DM and because of the short elimination half-life (t_1/2_) of metformin (5 h) [26]. In dose level −1, the everolimus dose was decreased rather than the metformin dose or the metformin dosing frequency because metformin pills <500 mg were not available and because of the difference in t_1/2_ between metformin (5 h) and everolimus (30 h) [26, 27]. In the event of intolerable toxicity at dose level 1, the exposure to the study treatment is probably decreased more effectively by decreasing the everolimus dose than the metformin dose or its dosing frequency.

**Table 1:** Dose-escalation schedule for everolimus and metformin. Metformin is given twice daily (the daily dose is double the shown dose)

Dose level	Everolimus (mg)	Metformin (mg, bid)
-1	5	500
1	10	500
2*	10	850
3*	10	1000
4*	10	1350
5*	10	1500
6*	10	1850

*Not reached in this dose-escalation study

### Pharmacokinetic analysis

To determine the pharmacokinetic interaction between everolimus and metformin, patients received (only for pharmacokinetic purposes) one single morning administration of metformin 1 day prior to start of treatment (day −1), at the dose level that the patient would receive at start of treatment. Blood samples were drawn on *t* = 0 (predose), 1, 2, 4, 6, 8 and 12 h after the morning doses of metformin on day −1 and 15. Blood samples for everolimus were drawn on day 7 (without metformin) and on day 15 (with metformin), both at predose and at 1, 2, 4, 6, 8, 12 and 24 h after administration of everolimus. Using these blood samples, full pharmacokinetic 12/24-h profiles of the metformin and everolimus plasma concentrations were determined, both as single agents and in combination with each other. Everolimus was determined in whole blood and metformin was determined in serum using high-performance liquid chromatography with tandem mass spectrometry (HPLC-MS/MS). According to conventional pharmacokinetic principles [28], the area under the curve (AUC) of the serum concentration curves was calculated as the AUC from *t* = 0 to infinity for the first administration of a study drug, i.e.*,* single-agent drug administration at day −1 for metformin, and as the AUC from *t* = 0 to t_last_ for steady-state drug administration, i.e.*,* at days 7 and 15 for everolimus (as single-agent and in combination with metformin, respectively) and at day 15 for metformin when it was combined with everolimus. T_last_ was the time point directly preceding the next dose for that agent, i.e.*,* 12 h for metformin bid and 24 h for everolimus qd.

### Evaluation of radiological tumor responses

Tumor responses were calculated as the relative difference between the volume of the target lesions at the radiological assessment during study inclusion and the radiological assessment that showed the best overall response. The cut-off marks of the total sum of the volume of the target lesions for progressive disease, stable disease and partial response were +20% and −30%, respectively. Complete response was defined as a disappearance of all target lesions.

### Statistical analysis

Descriptive statistics were used for evaluation of the adverse events, safety and efficacy of everolimus and metformin. The pharmacokinetic parameters were calculated using PKSolver [29]. All *P* values were calculated using the R statistical programming language (paired Student’s *t-*test).

## Results

### Characterization of the study cohort

In total, 12 patients were screened for eligibility between January 2013 and February 2014. Three patients did not meet the study inclusion criteria; one because of inadequate renal function, one because of inadequate bone marrow function, and one because of dysphagia. Table 2 summarizes the baseline characteristics of the nine eligible patients that were enrolled in the study. All patients had received one or more lines of treatment prior to study enrolment. Six patients received at least four weeks of study treatment and were thus evaluable for toxicity assessments (Table 3). Patients stayed on the study for a median duration of 48 days (range: 4–78). Two patients temporarily interrupted treatment: in one case due to trombocytopenia and in one case due to skin rash. Following treatment interruption, these patients received a 50% dose reduction of everolimus and metformin, respectively.

**Table 2:** Patient demographics and disease characteristics. Chemotherapy regimens were given in an advanced setting unless stated otherwise

Pt #	Gender	Age	WHO-PS	Primary diagnosis	Earlier surgery	Earlier chemotherapy	Earlier radiotherapy	Time since initial diagnosis (in years)
1	Male	68	1	Colorectal cancer	Yes, colostomy (palliative)	Irinotecan, capecitabine oxaliplatin, bevacizumab	No	1.28
2	Male	69	1	Colon cancer	Yes, hemicolectomy right side (curative)	Capecitabine (1 neoadjuvant course, 2 courses or advanced disease)	No	3.51
3	Male	57	1	Pancreatic cancer	No	Gemcitabine	Yes, advanced	0.92
4	Male	62	0	Pancreatic cancer	Yes, gastrojejunostomy (palliative)	Gemcitabine, erlotinib, FOLFIRINOX	No	2.38
5	Female	58	-	Vulvar cancer	Yes, vulvectomy with lymphadenectomy (curative)	Capecitabine	Yes, adjuvant	1.07
6	Female	51	1	Cervical cancer	Yes, lymph nodes debulking (curative)	Cisplatin (1 neoadjuvant course, 1 course for advanced disease), paclitaxel	Yes, adjuvant	1.82
7	Male	58	0	Gastric cancer	Yes, total gastric resection withesophago-jejunostomy and spleen extirpation (curative)	Erubiline, capecitabine, cisplatin (1 neoadjuvant course, 1 adjuvant course) Capecitabine, oxaliplatin, irinotecan	Yes, adjuvant	3.12
8	Male	55	1	Pancreatic cancer	No	FOLFIRINOX	No	1.05
9	Male	63	1	Cholangiocarcinoma	No	Gemcitabine, cisplatin	No	1.41

Abbreviations: *pt* patient, *WHO-PS* World Health Organisation performance status

**Table 3:** Description of administered doses, dose-limiting toxicities and serious adverse events. Patient #9 discontinued study treatment after 4 days on study due to toxicity. This is not shown in the table because the observed toxicity was due to everolimus monotherapy, not the study treatment combination of everolimus and metformin, as the patient discontinued study participation before the per-protocol start of metformin on day 8 of the study

Pt #	Everolimus dose (mg)	Metformin dose (mg)	DLT (grade)	SAE (grade)	Days on study	Reason for study termination	Overall survival (days after start of study)
1	10	500, bid	-	-	69	PD	114
2	10	500, bid	Thrombocytopenia (3)*	-	52	Toxicity	158
3	10	500, bid	Rash (3)**	-	78	Toxicity	229
4	10	500, bid	Thrombocytopenia (3)	Sepsis (4)	14	Toxicity	82
5	10	500, bid	-	-	48	PD	145
6	5	500, bid	-	Collapse (3)	10	Toxicity	83
7	5	500, bid	-	Bile duct stenosis (3), obstruction esophagus (3)	71	PD	184
8	5	500, bid	-	-	29	Toxicity	53

Abbreviations: *DLT* dose-limiting toxicity, *SAE* serious adverse event, *PD* progressive disease

*The everolimus dose of patient #2 was de-escalated to 5 mg after the DLT

**The metformin dose of patient #3 was de-escalated to 500 mg qd after the DLT

### MTD and DLTs

Nine patients started the study regimen consisting of one week of everolimus followed by the addition of metformin. Of the eight evaluable patients that received everolimus and metformin, five patients discontinued their study participation due to toxicity reasons and three patients because of progressive disease. Of the three patients who entered the study at the starting dose level of 10 mg everolimus qd and 500 mg metformin bid, one experienced a DLT (thrombocytopenia). The cohort receiving dose level 1 was then expanded, where after two more patients experienced a DLT (one case of thrombocytopenia and one case of skin rash). According to the protocol, the dose level was de-escalated to 5 mg everolimus qd and 500 mg metformin bid, at which no more DLTs were observed, but the toxicity of study treatment persisted (Tables 3 and 4). Subsequently, patient enrollment in the study was terminated. Regarding SAEs, one patient had to be hospitalized for a sepsis, one patient had to undergo surgery for a bile duct stenosis and obstructed esophagus (PTC drain and stent biliary duct, neo-gastric tube and stent) and one patient had to undergo an intervention for cholangitis and a liver abscess (PTC drain revised and abscess drainage).

**Table 4:** Possible, probable or definitively treatment-related adverse events

Dose level:	Dose level 1		Dose level −1	
Number of patients:	***n*** **= 6**		***n*** **= 3 (+2)**	
CTCAE grade:	**1–2**	**3–4**	**1–2**	**3–4**
*Hematological*				
Thrombopenia	3	2*		
Anemia	2			
Leucopenia	2			
Neutropenia	1			
*Clinical chemistry*				
Hyperbilirubinemia				1
Renal insufficiency	1			
*Clinical*				
Skin rash	1	1*		
Melena 1002		1		
Sepsis		1		
Epistaxis	3			
Fatigue	3			
Anorexia	2			
Cough	2			
Dry skin	1		(1)	
Mucositis 1001	2			
Nausea	1			
Stomatitis	1		(1)	
Blood in stool 1002	1			
Diarrhea 1002	1			
Erythema	1			
Gingival bleeding	1			
Hand-foot syndrome	1			
Paronychia	1			
Pruritus	1			
Swollen tongue			(1)	
Weight loss	1			

Numbers represent number of patients. Numbers between brackets at the −1 dose level represent patients that experienced adverse events after intrapatient dose de-escalation from dose level 1 to dose level −1

Abbreviations: *CTCAE* Common Terminology Criteria for Adverse Events version 3.0

*Indicates that the adverse event was also a DLT

### Safety

The treatment-related CTC-graded adverse events per dose level are listed in Table 4. Regarding severe (grade 3/4) adverse events, one patient with a grade 4 sepsis, two patients with grade 3 thrombocytopenia, one patient with a grade 3 rash, one patient with a grade 3 melena and one patient with a grade 3 hyperbilirubinemia were observed. The most frequently reported clinical toxicities of any grade included epistaxis, fatigue (both 33%), anorexia, coughing, dry skin, mucositis, skin rash and stomatitis (all 22%). The combination of everolimus and metformin did not induce significant changes in hematological or clinical chemistry parameters. Interestingly, glucose and insulin levels increased instead of decreased during study treatment (**Supplementary Table** **1**).

### Pharmacokinetics

**Fig. 1:**
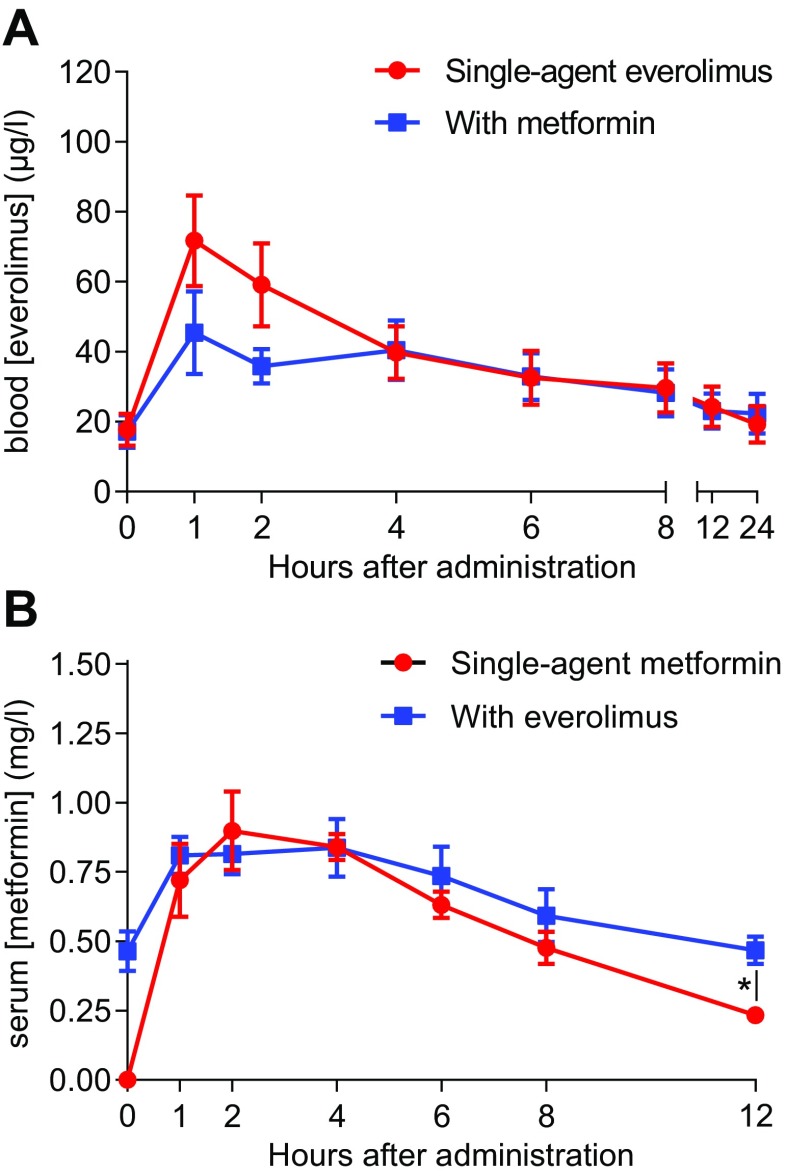
Concentration curves of everolimus and metformin when administered as single-agent and in combination. **a** Mean ± SEM of the blood everolimus concentration-time curves in μg/l of 5 evaluable patients who received 10 mg everolimus qd. **b** Mean ± SEM of the serum metformin concentration-time curves in mg/l of 7 evaluable patients who received 500 mg metformin bid. Everolimus and metformin levels were determined in whole blood and in serum, respectively, by high-performance liquid chromatography with tandem mass spectrometry (HPLC-MS/MS). The maximum concentration levels in the figure do not necessary correspond with the C_max_ as described in Table 5, because not all patients had their C_max_ at the same time point

Concentration-time curves of everolimus and metformin, both as single agents and in combination, were obtained from seven patients and are shown in Fig. 1 **.** The observed pharmacokinetic parameters for patients with complete concentration time-curves are shown in Table 5 and **Supplementary Table** **2** **.** An increased everolimus clearance was observed when it was administered in combination with metformin than as single agent (clearance: 0.018 l/h vs. 0.0097 l/h; *P* = 0.0018), but this did not affect everolimus exposure (area under the curve, AUC). No difference in clearance was detected between metformin administration as single agent or in combination with everolimus (61.1 l/h vs. 59.9 l/h, *P* = 0.86). Nevertheless, the t_1/2_ and elimination rate constant (λ_z_) of metformin were increased when co-administered with everolimus as compared with single-agent administration (t_1/2_: 7.95 h vs. 4.16 h; *P* = 0.017 and λ_z_: 0.17 h^−1^ vs. 0.09 h^−1^; *P* = 0.005). The metformin AUC was comparable between single-agent administration and co-administration with everolimus. There was no association between the extent of pharmacokinetic interaction between everolimus and metformin and the occurrence of toxicity in the individual patients. Three patients (#5, #8 and #9) had a higher exposure to metformin when administered in combination with everolimus than as single agent due to slower drug elimination (**Supplementary Figs.** **1** **and 2**), but patients #5 and #8 experienced only minor toxicity and discontinued study treatment due to progressive disease. Although patients #8 and #9 had incomplete concentration-time curves for metformin, they had an evidently increased metformin exposure when administered in combination with everolimus. However, due to the incompleteness of their data, they could not be included in the quantitative pharmacokinetic analyses shown in Table 5.

**Table 5:** Pharmacokinetic parameters of everolimus and metformin when administered as single-agent and in combination. Data are mean (SD)

Parameter	Unit	Everolimus 10 mg qd (*n* = 5)			Metformin 500 mg bid (*n* = 5)		
		Single-agent	In combination	*P* value	Single-agent	In combination	*P* value
C_max_	μg/l	73.1 (28.5)	53.3 (20.7)	0.18	912 (227)	1027 (261)	0.31
T_1/2_	h	23.1 (7.1)*	23.2 (5.5)*	0.95	4.16 (0.44)	7.95 (2.03)	0.017
λ_z_	h^−1^	0.028 (0.006)	0.029 (0.006)	0.77	0.17 (0.02)	0.09 (0.02)	0.005
AUC	mg/l*h	0.713 (0.35)	0.639 (0.30)	0.50	8.32 (1.18)	8.12 (1.87)	0.78
CL/F	mg/(mg/l)/h	0.0097 (0.004)*	0.018 (0.006)*	0.0018	61.1 (9.0)	59.9 (14.7)	0.86
V_z_/F	mg/(mg/L)	0.30 (0.004)*	0.34 (0.15)*	0.13	366 (60)	461 (15)	0.16

Abbreviations: *C*_*max*_ maximal concentration, *t*_*1/2*_ *,* elimination half-life, *λ*_*z*_, elimination rate constant, *AUC* area under the curve, *CL* clearance, *F* systemic availability of the administered dose, *V*_*z*_ apparent volume of distribution during terminal phase

*T_1/2_, CL and V_z_ are pharmacokinetic parameters that are independent of dose and were calculated for all 7 patients with everolimus concentration-time curves. The 24 h time point of patient #3 was calculated using extrapolation of the 6 h, 8 h and 12 h time points on a logarithmic scale. *P* values were calculated using paired Student’s *t-*test. Pharmacokinetic parameters for 2 patients who received 5 mg everolimus qd and 500 mg metformin bid are shown in Supplementary Table 2

### Tumor responses

After nine weeks of treatment, three patients were still on study and all had stable disease, with tumor responses of −14%, +3% and −5% of the volume of all target lesions. The three patients that received nine weeks of treatment had a longer survival than the five patients who had to discontinue study treatment prematurely due to toxicity (median overall survival: 184 days vs. 82 days; *P* = 0.0482, Table 3). Of the three patients that received nine weeks of treatment, two eventually discontinued study treatment due to progressive disease and one because of toxicity reasons (Table 3). The nine-week time point for this survival analysis was prespecified because patients were to receive a CT scan to evaluate tumor responses at this time point.

## Discussion

This study explored the safety and pharmacokinetics of the combination of everolimus and metformin in patients with advanced solid malignancies. We found that a combination regimen of everolimus and metformin is poorly tolerated in these patients. Therefore, we were unable to determine the MTD for this combination treatment.

Recently, a retrospective study of 31 patients with pancreatic neuroendocrine tumors (PNETs) was published [30]. This study showed improved clinical outcomes to treatment with everolimus and octreotide in patients that were also treated with metformin for diabetes as compared with patients that were treated with insulin or as compared with nondiabetic patients. In contrast to our study, Pusceddu et al. [30] made no mention of intolerable toxicity. By speculation, possible explanations for this apparent difference come to mind. First, diabetic PNET patients that were already being treated with metformin may better tolerate everolimus than cancer patients that are treated with metformin and everolimus for the first time because the former group may have had more time to habituate to metformin. Second, metformin was administered 500 mg bid upfront in our study, whereas the patients described by Pusceddu et al. received metformin after a titration period, starting at 500 mg qd in the first week [30]. The latter approach mimics metformin treatment schedules in T2DM and decreases the initial side effects of metformin, especially those of gastro-intestinal nature [31, 32]. What argues against this possible explanation for metformin toxicity is that only two patients of the present study experienced gastro-intestinal side-effects and all gastro-intestinal side-effects were grade 1–2, except one case of grade 3 melaena. Third, octreotide might ameliorate the toxic effects of the combination regimen of everolimus and metformin through mechanisms that are still unknown. Fourth, a retrospective study design such as that of Pusceddu et al. might lead to unintentional under-ascertainment of toxicity when side-effects lead to discontinuation of treatment, which prevents the inclusion of these patients in a retrospective analysis.

In a phase I clinical trial, Khawaja et al. investigated the combination of metformin and another mTOR inhibitor, the intravenously administered temsirolimus [14]. The authors concluded that this combination was well tolerated with only two DLTs observed among 21 patients and an MTD/recommended dose of 25 mg temsirolimus weekly and 1000 mg metformin bid. In contrast, MacKenzie et al. observed severe toxicity in a similar phase I clinical trial in patients with advanced cancers that were also treated with metformin and temsirolimus, reporting an MTD of 20 mg temsirolimus weekly and 500 mg metformin qd [33]. The main difference between the two study designs was that the former utilized a titration period for metformin to limit its toxicity, whereas the latter did not.

On one hand, altered pharmacokinetics of everolimus were observed when everolimus was administered in combination with metformin as compared with everolimus single-agent administration. While the maximal everolimus concentration was lower in combination than as single-agent, this was statistically not significant but still might suggest that metformin affects everolimus absorption. Furthermore, everolimus clearance was significantly higher in combination with metformin than as single-agent, while the elimination half-life was unchanged, possibly due to a moderate, but unsignificant, increase in the volume of distribution.

On the other hand, altered metformin pharmacokinetics were observed when metformin was administered in combination with everolimus as compared with metformin single-agent administration, i.e. a lower elimination half-life and elimination rate constant. However, the significantly slower metformin elimination was not accompanied by a significantly slower clearance. This might be explained by a moderate, but unsignificant, increase in the volume of distribution. The slower metformin elimination did not translate to higher AUCs in combination with everolimus versus single-agent administration, which suggest that the observed toxicity of the study treatment cannot be explained by higher metformin AUCs in combination with everolimus versus single-agent administration. A limitation of our pharmacokinetic analysis is that two patients who had an evidently increased exposure to metformin due to slower drug elimination had incomplete time-concentration curves and could thus not be included in our pharmacokinetic analysis. This may explain why the quantitative pharmacokinetic analysis did not show a higher metformin AUC when administered in combination with everolimus relative to single-agent administration. Alternatively, our results may suggest that pharmacodynamic rather than pharmacokinetic interactions between everolimus and metformin explain the high toxicity of this drug combination. To the best of our knowledge, there is no data on pharmaco-interactions between everolimus and metformin at the clinical or molecular level yet.

The combination of everolimus and metformin was tolerated for at least nine weeks in three patients and they all had stable disease. Patients treated with everolimus and metformin for at least nine weeks had a prolonged survival compared with those who had to discontinue study treatment prematurely due to toxicity, but these analyses were conducted in very small groups and should be carefully considered as merely preliminary evidence of anti-tumor efficacy of the combination of an mTOR inhibitor and a biguanide. Another study, investigating a combination of metformin and the mTOR inhibitor temsirolimus, also observed modestly promising anti-tumor efficacy in a cohort with heavily pretreated patients with advanced cancer [14].

In conclusion, results from a prospective, open-label, single-center phase I study show that the combination of everolimus and metformin is poorly tolerated in patients with advanced cancer. This may be due to pharmaco-interactions between the two drugs, because everolimus delayed and inhibited the elimination of metformin. Our findings have implications for daily practice, especially for diabetic patients using metformin that also have a cancer being treated with everolimus. In addition, our data may be important for the interpretation and design of ongoing and future clinical trials that study the combination of everolimus and metformin (see ClinicalTrials.gov entries NCT01627067, NCT01797523 and NCT02294006). Although our analyses of tumor responses and overall survival are based on very small groups and should be interpreted with the greatest caution, they could suggest that combining an mTOR inhibitor and a biguanide has anti-cancer activity in patients with advanced cancer. Therefore, alternative combination regimens [14] could be investigated in future studies with drugs other than metformin and/or everolimus.

## Electronic supplementary material

ESM 1 (PDF 691 kb)
