# Supplementary material for: A phase Ib study of everolimus combined with metformin for patients with advanced cancer
Source: Invest New Drugs. 2017 Jun 15;36(1):53–61. doi: 10.1007/s10637-017-0478-4 (PMC5805805; doi:10.1007/s10637-017-0478-4)
Supplement: Supplementary file 1 — (PDF 691 kb) [file 10637_2017_478_MOESM1_ESM.pdf]

SUPPLEMENTARY FIGURES

Supplementary Figure 1:

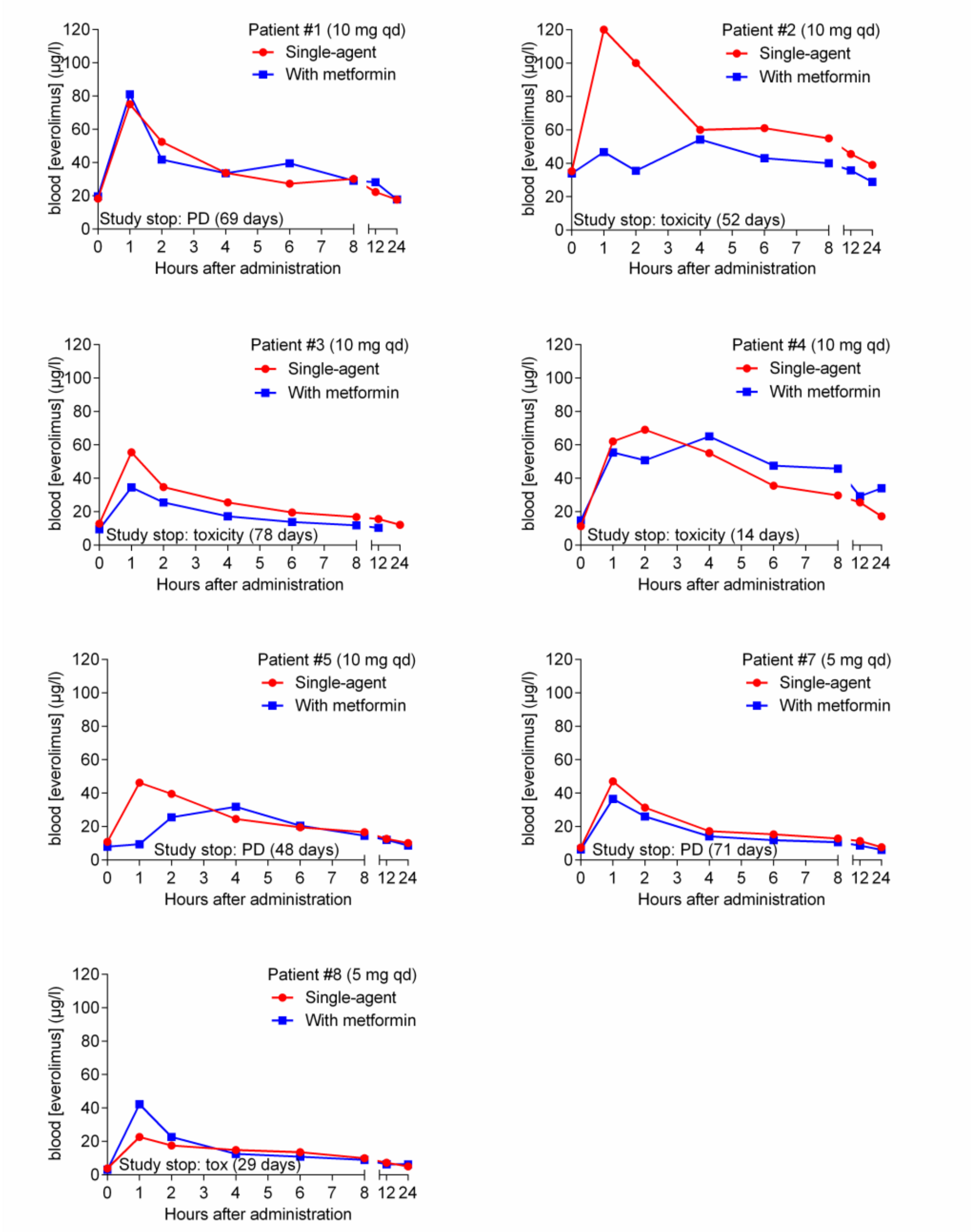

Everolimus concentration-time curves for individual patients. Blood everolimus concentration-time curves in µg/l of 5 patients who received 10 mg everolimus qd (#1-5) and 3 patients who received 5 mg everolimus qd (#6-8). Everolimus levels were determined in whole blood by high-performance liquid chromatography with tandem mass spectrometry (HPLC-MS/MS). The duration on study treatment and reason for study termination is shown for each patient. Abbreviations: PD, progressive disease; tox, toxicity.

Supplementary Figure 2:

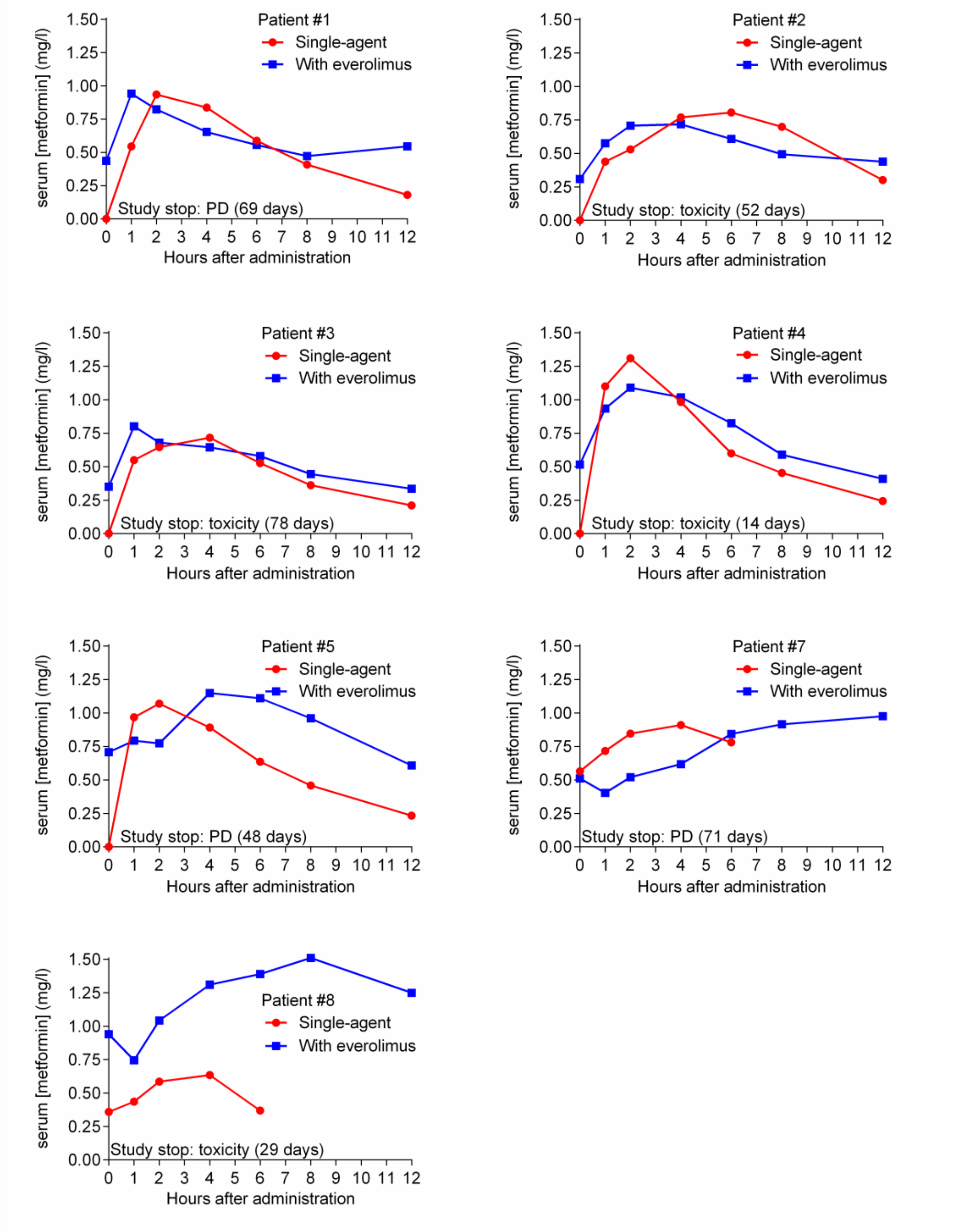

Metformin concentration-time curves for individual patients. Serum metformin concentration-time curves in mg/l of 9 patients who received 500 mg metformin bid. Metformin levels were determined in serum by high-performance liquid chromatography with tandem mass spectrometry (HPLC-MS/MS). The duration on study and reason for study termination is shown for each patient. Abbreviation: PD, progressive disease.

SUPPLEMENTARY TABLES

Supplementary Table 1:

Lab test	Screening (*n*)	Day 1 (*n*)	Day 22 (*n*)	Day 43 (*n*)	Day 64 (*n*)
**Hemoglobin (mmol/l)**	7.6 (9)	7.2 (6)	7.2 (7)	7.3 (6)	6.8 (3)
**Leukocytes (*10^9/l)**	7.8 (9)	8.4 (5)	6.7 (7)	7.2 (6)	6.1 (3)
**Neutrophils (*10^9/l)**	5.2 (9)	6.6 (5)	4.5 (6)	5.2 (6)	4.3 (3)
**Platelets (*10^9/l)**	243.2 (9)	207 (5)	160.4 (7)	196.7 (6)	198 (3)
**Sodium (mmol/l)**	138.3 (9)	138.5 (4)	138 (3)	134.8 (6)	135.7 (3)
**Potassium (mmol/l)**	3.8 (9)	3.9 (5)	3.6 (3)	3.9 (6)	3.8 (3)
**Creatinin (µmol/l)**	78.8 (9)	68 (5)	87 (3)	78.7 (6)	75 (3)
**Ureum (mmol/l)**	4.4 (9)	4.6 (4)	5.2 (3)	4.7 (6)	4.1 (3)
**Albumin (g/l)**	41.7 (9)	39 (5)	41 (2)	39.2 (5)	38.3 (3)
**CRP (mg/l)**	30.7 (8)	24.5 (4)	6.2 (1)	47.0 (5)	78.3 (3)
**Glucose (mmol/l)**	5.5 (9)	6.5 (6)	7.6 (2)	9.5 (5)	14.2 (3)
**Insulin (pmol/l)**	112.1 (8)	101.5 (2)	164.5 (2)	137.5 (4)	230.7 (3)
**IGF-bind. prot.-3 (mg/l)**	1.6 (8)	2.0 (2)	1.6 (2)	1.6 (4)	1.5 (3)
**IGF-1 (nmol/l)**	18.6 (8)	34 (1)	15 (2)	18.3 (4)	17.3 (3)

Hematology and clinical chemistry parameters for included patients during study treatment.

Supplementary Table 2:

Parameter	Unit	Everolimus (5 mg, n = 2)	
		Single-agent	In combination
**C_{max}**	µg/l	34.80	39.45
**λ_z**	h^{-1}	0.044	0.035
**AUC**	µg/l*h	281.78	253.61

Pharmacokinetic parameters of 5 mg everolimus qd (n = 2 patients). $T_{1/2}$, CL and V_z are pharmacokinetic parameters that are independent of dose and were calculated for all 7 patients with everolimus concentration-time curves and are shown in **Table 5** of the main manuscript.
